# Silver Pomfret (*Pampus argenteus*) Aquaculture: Advances, Bottlenecks, and Future Strategies

**DOI:** 10.3390/ani15223347

**Published:** 2025-11-20

**Authors:** Guangde Qiao, Bingfei Li, Qiaozhen Ke, Shunshun Tao, Wantu Xu, Yabing Wang, Shiming Peng

**Affiliations:** 1 East China Sea Fisheries Research Institute, Chinese Academy of Fishery Sciences, Shanghai 200090, China; 2 Xiangshan Harbor Aquatic Seedling Co., Ltd., Ningbo 315702, China

**Keywords:** *Pampus argenteus*, aquaculture development, industrialization, sustainable aquaculture

## Abstract

Silver pomfret (*Pampus argenteus*) is an economically valuable marine species with growing potential for large-scale aquaculture in China. In recent years, significant progress has been achieved in artificial breeding, nutritional research, and disease management; however, the industry’s development remains constrained by technological and operational challenges. This review provides an overview of the biological characteristics of *P. argenteus* and systematically summarizes the research progress and technological applications in artificial breeding, nutritional physiology, disease management, and aquaculture systems. It also analyzes the key constraints hindering the development of its aquaculture industry. Based on current research progress and industry needs, five strategic measures are proposed to accelerate industry development: (1) establishing a comprehensive germplasm repository; (2) breeding high-performance new varieties; (3) developing species-specific compound feeds; (4) enhancing disease prevention and biosecurity systems; and (5) standardizing operational protocols for different aquaculture systems. The implementation of these measures will provide a theoretical reference and technical support for the high-quality development of the *P. argenteus* aquaculture industry in China.

## Simple Summary

Silver pomfret (*Pampus argenteus*) is a valuable marine fish widely consumed in China because of its tender meat and high nutritional quality. In recent years, researchers have made great progress in artificial breeding and aquaculture techniques for this species. However, the overall scale of *P. argenteus* aquaculture is still small, and several technical and management challenges remain. This review introduces the main biological features of *P. argenteus* and summarizes the latest research progress in breeding, nutrition, disease control, and aquaculture systems. It also identifies the key problems that limit industry expansion and proposes five major strategies to promote sustainable growth, including improving germplasm resources, developing new varieties, optimizing feeds, strengthening disease prevention, and standardizing operational protocols. These efforts aim to support the healthy and efficient development of the *P. argenteus* aquaculture industry in China.

## 1. Introduction

Silver pomfret (*Pampus argenteus*), a species belonging to the genus *Pampus* within the order Perciformes and the family Stromateidae, is widely distributed in China’s coastal waters, including the Bohai Sea, Yellow Sea, East China Sea, and South China Sea (Table 1) [1]. As a commercially valuable marine species, *P. argenteus* has attracted considerable attention due to its excellent nutritional profile and high market value. Since the last century, researchers have conducted extensive research on its biological characteristics, artificial breeding, larval rearing, and aquaculture [2]. With the ongoing transformation and modernization of China’s marine fisheries, *P. argenteus* is poised to become an increasingly valuable species for mariculture diversification in China. In recent years, significant progress has been made in areas such as artificial breeding, germplasm selection, broodstock domestication, larval rearing technology, feed formulation, breeding strategies, and disease control. However, compared with other major marine aquaculture species such as large yellow croaker (*Larimichthys crocea*, 292,615 t in 2024) and golden pomfret (*Trachinotus ovatus*, 305,552 t in 2024) [3], the industrial development of *P. argenteus* is still in its infancy.

Issues such as inconsistent larvae quality, low breeding efficiency, slow growth rates, poor disease resistance, and low survival rates remain prominent. Additionally, the lack of high-quality germplasm resources, standardized aquaculture protocols, and comprehensive health management systems continues to hinder the scaling up and sustainable development of the industry. Therefore, it is of great significance to thoroughly review the progress in *P. argenteus* aquaculture research, identify key technical bottlenecks, and define priority areas for technological innovation to promote the sustainable development of the aquaculture industry. This review aims to summarize recent advances in the understanding of *P. argenteus* biological characteristics, artificial breeding, and aquaculture technologies. It also highlights the major obstacles limiting industrial development and proposes targeted solutions and development strategies based on emerging technologies and practical production needs. The goal is to provide a theoretical foundation and technical guidance for fostering the high-quality development of the *P. argenteus* aquaculture industry.

## 2. Biological Characteristics of *P. argenteus*

*P. argenteus* is a typical warm-temperate, nearshore pelagic fish species with highly specialized morphological, physiological, and ecological adaptations [4]. The body is ovoid and strongly laterally compressed, with the greatest depth at the dorsal fin origin. The caudal peduncle is short and slender, the head small, the snout rounded, and the mouth small and subterminal. Both jaws bear a single row of fine, closely set tricuspid teeth, an adaptation for pecking on small plankton. No teeth are present on the vomer, palatine bones, or tongue. The body is covered with small, thin deciduous cycloid scales. This feature becomes particularly evident under stress, complicating handling in aquaculture and increasing the risk of secondary infections. The lateral line is well-developed, positioned entirely on the upper side of the body, and runs to the caudal fin base. Its intricate dorsal and ventral branches enhance sensitivity to water flow changes, facilitating rapid evasion from predators. The fin morphology is distinctive: in adults, the pointed, elongated pectoral fins may reach up to half the body length; the caudal fin is deeply forked; and the anal and dorsal fins are nearly opposite, a combination that supports sustained swimming. Body coloration varies with developmental stage: juveniles have stellate melanophores scattered along the body sides, while in adults, they are mainly concentrated anteriorly. The dorsal surface is bluish-gray, the ventral side silvery-white, and all fins are light gray [5].

**Figure 1:**
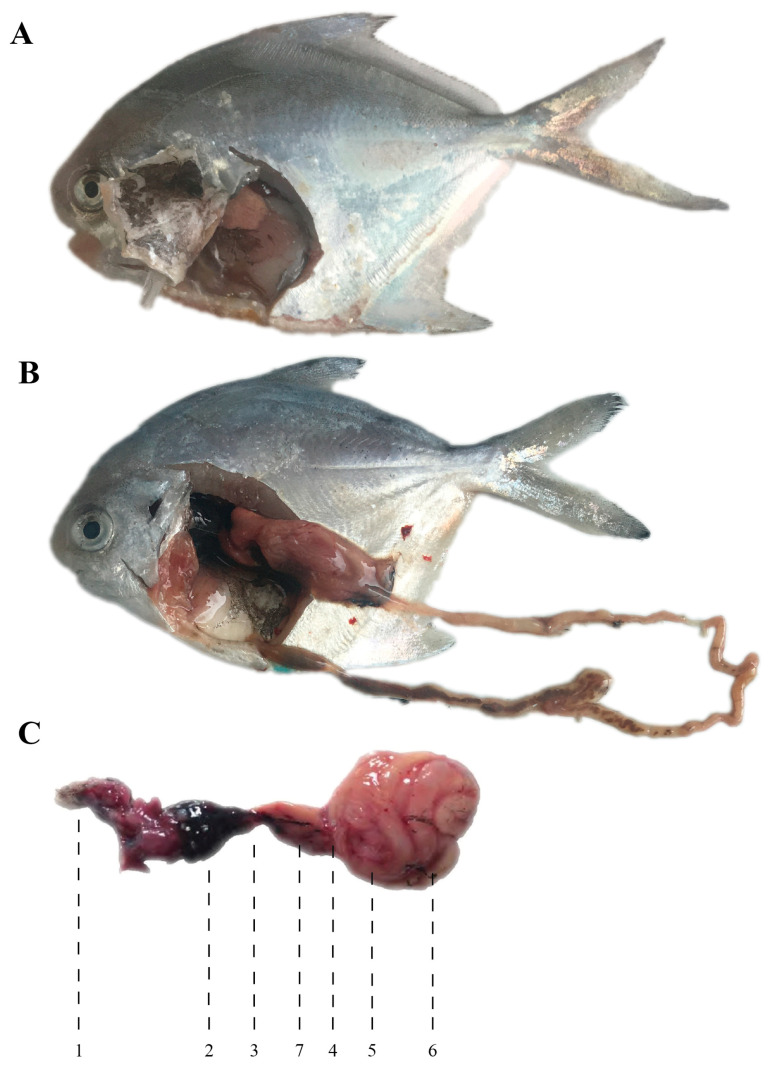
Internal structure of *Pampus argenteus*. (**A**,**B**): anatomical diagrams; (**C**): digestive systeanatomy [7]. 1: Tongue; 2: Pharyngeal sac; 3: Esophagus; 4: Stomach; 5: Pyloric caeca; 6: Intestine; 7: Liver.

Internally, the most distinctive features of *P. argenteus* are the absence of a swim bladder and the presence of oval pharyngeal sac, a characteristic trait of the suborder Stromateoidei (Figure 1) [6,7,8]. The lack of a swim bladder necessitates constant swimming to maintain buoyancy, resulting in an exceptionally high demand for dissolved oxygen. The liver is bilobed, and the pancreas is distinct, extending along the intestinal tract—adaptations that support a diet rich in protein and lipids [5].

Ecologically, *P. argenteus* is eurythermal and euryhaline, but its tolerance ranges have strict thresholds (Table 2) [5]. In its natural habitat, it inhabits waters with temperatures of 8–26 °C, while under aquaculture conditions, the optimal range is 25–28 °C. Feeding activity decreases significantly below 14 °C or above 32 °C, and beyond these thresholds, mortality may occur [5]. The species tolerates salinities of 10–36, with an optimum of 20–25. Notably, gonadal development depends on relatively high salinity. Sudden salinity changes or prolonged exposure to low salinity can impair gill filament osmoregulatory function, leading to mortality. Light conditions also significantly influence behavior, as intense light can induce stress and leaping, exacerbating scale loss.

*P. argenteus* is a generalist feeder, consuming crustaceans, jellyfish, arrow worms, and cephalopod larvae, with a notable preference for jellyfish [9,10,11,12]. Studies have shown that its jellyfish ingestion can exceed ten times its body weight [13], and stable isotope analysis confirms jellyfish as the primary dietary contributor [14]. Reproductive studies indicate early gonadal development in this species [15]. In females, ovarian differentiation begins at approximately 68 days post-hatching (dph), while in males, testicular differentiation occurs around 108 dph under controlled aquaculture conditions (water temperature 26 ± 1 °C; salinity 25; dissolved oxygen 6.5 ± 1.5 mg/L; pH 7.9 ± 0.2) [16]. The minimum age of sexual maturity is approximately one year, with the vast majority of individuals reaching maturity by age two. Spawning grounds are mainly located in estuarine brackish water mixing zones [5]. The spawning period occurs progressively later from south to north, and an autumn spawning cohort exists in some regions [5]. Overall, the reproductive strategy of *P. argenteus* is characterized by early sexual maturity, batch spawning, and concentrated spawning grounds.

## 3. Germplasm Resource Characteristics of *P. argenteus*

### 3.1. Cytogenetics

**Figure 2:**
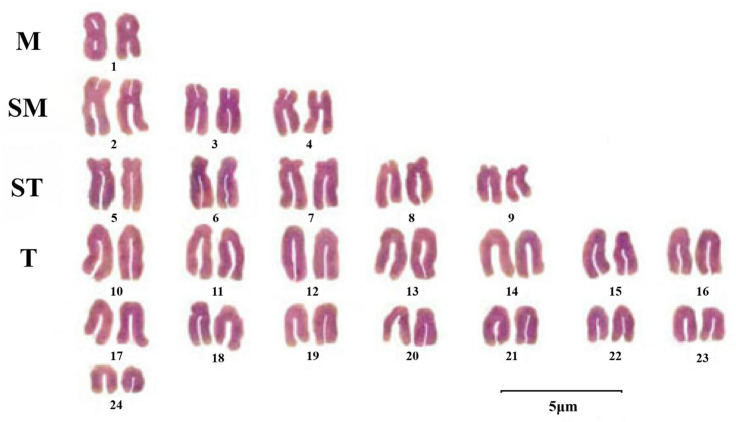
Karyotype of *P. argenteus* (scale bar = 5 μm) [17]. M: Metacentric; SM: Submetacentric; ST: Subtelocentric; T: Telocentric.

Cytogenetic studies have revealed that *P. argenteus* possesses a distinct and stable karyotype. Studies employing intraperitoneal injections of PHA and colchicine, coupled with the direct kidney cell preparation method, consistently report a diploid chromosome number of 2n = 48 (Figure 2) [17]. The karyotype formula is 2n = 2 M + 6 SM + 10 ST + 30 T, with a fundamental arm number (NF) of 56 [5]. This pattern, dominated by telocentric chromosomes, is characteristic of derived teleost groups within the Perciformes and contrasts with the primitive groups like Cypriniformes and Siluriformes, which have over 50% metacentric (M) chromosomes, supporting the derived phylogenetic status of *P. argenteus* [18]. While minor variations in karyotypic formulae (e.g., 2n = 2 SM + 10 ST + 36 T, NF = 50) have been reported, these are attributable to differences in chromosome condensation during preparation and observational criteria [19]. The conserved diploid number and overall karyotypic architecture confirm the stability of its cytogenetic characteristics.

### 3.2. Biochemical Genetics

Biochemical genetic analysis of five tissues (spleen, liver, muscle, heart, kidney) via polyacrylamide gel electrophoresis was conducted on four key isozymes, which generated a total of 61 bands (Table 3) [20]. Among them, esterase (EST) displayed the highest complexity with 30 bands, followed by lactate dehydrogenase (LDH, 16 bands), glutamate dehydrogenase (GDH, 9 bands), and alcohol dehydrogenase (ADH, 6 bands). This variation in band number reflects the functional diversity of each enzyme. Tissue-specific expression patterns were evident: the liver, as the primary metabolic organ, showed the highest band count (18) and intensity, followed by the heart (15 bands) and muscle (12 bands), correlating with their respective energy demands for physiological activity and locomotion. The spleen exhibited minimal activity (5 bands), consistent with its primary immune function. Distinct isozyme patterns were observed: EST activity was strongest in the liver and heart; the full LDH1–LDH5 spectrum was liver-specific; GDH4 was universally active; and ADH2 was liver-specific. This tissue-specific isozyme profile reflects genetic adaptations to its pelagic lifestyle and provides potential biomarkers for germplasm assessment.

### 3.3. Molecular Genetics and Genomics

**Figure 3:**
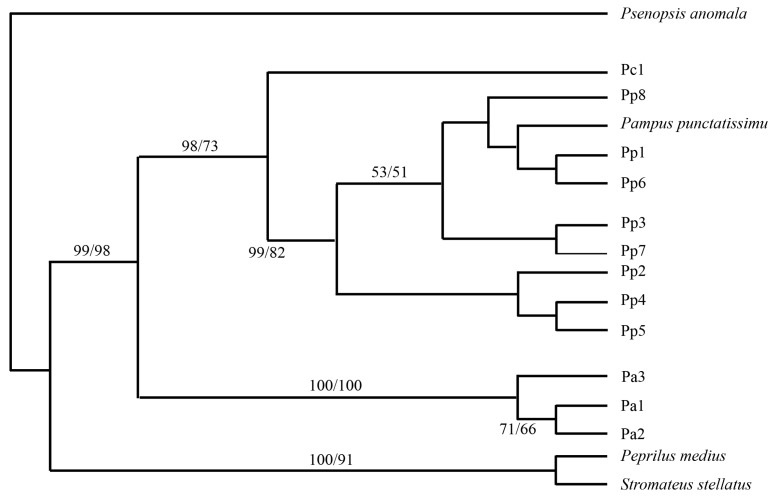
Construction of phylogenetic tree in the *Pampus* genus based on mitochondrial *COI* gene fragment (numbers indicate bootstrap confidence values of Neighbor-Joining (NJ) and Maximum Likelihood (ML) methods) [21]. Pa: *P. argenteus*, Pc: *P. chinensis*, Pp: *P. punctatissimus*.

**Figure 4:**
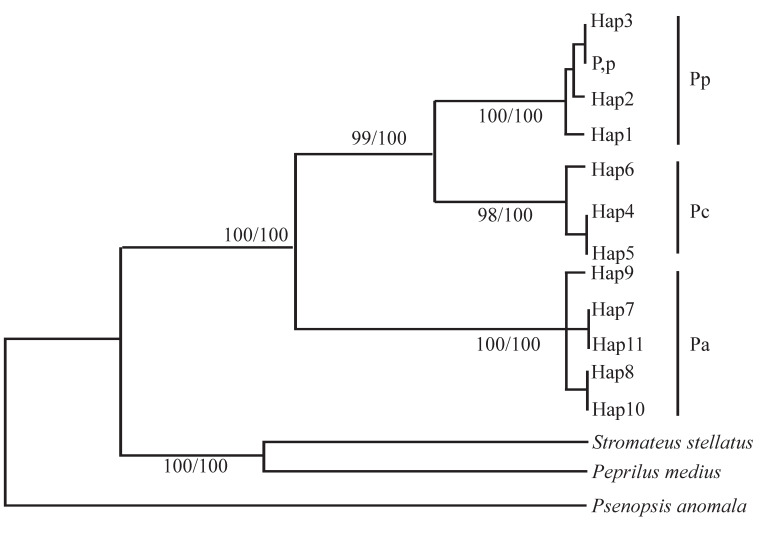
Construction of phylogenetic tree in the *Pampus* genus based on mitochondrial *Cyt-b* gene fragment (numbers indicate bootstrap confidence values of NJ and ML methods) [22]. Pa: *P. argenteus*, Pc: *P. chinensis*, Pp: *P. punctatissimus*, Hap: haplotype.

Significant genetic divergence exists between *P. argenteus* and its congeners, *P. punctatissimus* and *P. chinensis*, as revealed by mitochondrial COI (603 bp) and *Cyt-b* (1123 bp) gene sequences [21,22]. For the COI, the genetic distance between *P. argenteus* and *P. punctatissimus* ranges from 0.151 to 0.162, while that between *P. argenteus* and *P. chinensis* is 0.165–0.168. In contrast, the distance between *P. punctatissimus* and *P. chinensis* is only 0.058–0.065. From the phylogenetic tree, the eight *COI* haplotypes of *P. punctatissimus* clustered together with the *P. punctatissimus COI* sequences downloaded from GenBank, forming a single branch. This branch further grouped with *P. chinensis*, while the three *COI* haplotypes of *P. argenteus* formed another distinct branch (Figure 3). For *Cyt-b*, the divergence between *P. argenteus* and *P. punctatissimus* is the highest among the three (0.108). Phylogenetically, haplotypes of *P. argenteus* form a distinct clade, with *P. punctatissimus* and *P. chinensis* clustering together, indicating that *P. argenteus* is the earliest diverging species within the genus (Figure 4).

Analysis of the mitochondrial D-loop region in three wild populations (Bohai Sea, East China Sea, and South China Sea) reveals high haplotype diversity (average Hd = 0.88) but low nucleotide diversity (average π = 0.006), a pattern often associated with historical population expansion. This is supported by a unimodal mismatch distribution and significantly negative values in neutrality tests (Tajima’s D = −1.106, *p* = 0.138; Fs = −26.601, *p* = 0.000), confirming a past expansion event in the East China Sea population [23]. Genetic differentiation analysis shows that 94.99% of variation occurs within populations, while significant differentiation exists among populations (Fst = 0.050, *p* < 0.05). Pairwise Fst values are significant between the South China Sea and both the Bohai Sea (Fst = 0.09) and East China Sea (Fst = 0.08) populations, but not between the Bohai and East China Sea populations, likely due to gene flow facilitated by the Yellow Sea–East China Sea current system [24].

AFLP marker analysis indicates significant genetic differentiation among six populations from the Yellow Sea and East China Sea (average Fst = 0.11, *p* < 0.001) [25]. Sun [26] employed 13 inter simple sequence repeat (ISSR) primers to assess the genetic diversity of three cultured generations and one wild population of *P. argenteus*. The percentages of polymorphic loci were 85.50%, 79.71%, 83.33%, and 76.81%, respectively. Although the cultured populations exhibited slightly lower polymorphism levels than the wild population, the overall proportion of polymorphic loci remained high, indicating that *P. argenteus* still maintains a relatively good level of genetic diversity. Transcriptome sequencing has identified 107,007 Simple sequence repeat (SSR) loci from 97,289 UniGenes, predominantly mono- and dinucleotide repeats (82.24%) [27]. Notably, 76.95% of these SSRs have repeat lengths > 12 bp, indicating high polymorphism potential for genetic linkage mapping and QTL analysis. A milestone was reached in 2024 with the assembly of a high-quality chromosome-level genome (size: 518.06 Mb; contig N50: 20.47 Mb; scaffold N50: 22.86 Mb), containing 24,696 protein-coding genes and achieving 98.9% BUSCO completeness [28]. Subsequent studies have produced improved, sex-specific chromosome-level assemblies, providing invaluable resources for genetic improvement and evolutionary studies [29].

## 4. Development Process of Artificial Breeding Technology

### 4.1. Overseas Research Progress

Since the 1960s, research on artificial breeding techniques for *P. argenteus* has been initiated in several countries. Japanese scholar Mito [30] conducted the first artificial insemination experiments in Japan’s Seto Inland Sea and systematically documented embryonic and larval development. In the 1990s, researchers in Kuwait initiated studies on the reproductive biology of *P. argenteus* in the Arabian Gulf. Dadzie et al. [31,32] identified the spawning season and reproductive characteristics of *P. argenteus* in Kuwaiti waters, while Almatar et al. [33] further quantified spawning frequency, fecundity, egg quality, and spawning pattern. In larval rearing, Al-Abdul-Elah et al. [34] improved the survival rate of 38-day-old larvae from 1.5% to 3.6–4.2% through optimizing feed. Building on this foundation, Kuwait successfully produced offspring from artificially cultured broodstock for the first time in 2007, although the survival rate remained low (3–4%) [35]. In recent years, research in Kuwait has shifted toward compound feeds [36] and nutritional composition analysis [37], yet no major breakthrough has been achieved in improving larvae survival. Studies indicate that high mortality in early larvae stages is a primary constraint. Azad et al. [38] reported that cumulative larvae mortality exceeded 50% within 24 days post-hatching. Between 2012 and 2016, research by the Kuwait Institute for Scientific Research revealed low hatching rates of fertilized eggs and poor larval survival from wild broodstock. Although artificially bred broodstock produced large quantities of eggs, fertilization rates were low and larvae survival was brief. It was suggested that mortality was linked to inferior gamete quality and that broodstock management techniques require further refinement [39]. Despite decades of research, progress in Kuwait and other countries has remained constrained by poor broodstock performance, unstable fertilization rates, and low larval survival. The absence of advanced environmental control systems and refined feeding strategies has prevented large-scale success. To better illustrate these differences, Table 4 summarizes the overseas progress in artificial breeding of *P. argenteus*, highlighting survival rates.

### 4.2. Domestic Research Progress

In the late 1980s, Zhao et al. pioneered research on the reproductive biology of *P. argenteus* in China, providing the first descriptions of eggs and larvae morphology in the East China Sea. However, due to the significant challenges in artificial breeding, although extensive studies were conducted by the end of the 20th century, no successful cases had been reported by the year 2000. Over the past two decades, China has achieved a series of advances in the artificial breeding and aquaculture of *P. argenteus*. In 2004, the East China Sea Fisheries Research Institute of the Chinese Academy of Fisheries Sciences initiated research on artificial breeding and successfully produced the first batch of larvae in 2006, achieving a survival rate of 14.1% [5]. In subsequent years, the research team addressed key challenges such as active swimming behavior and weak feeding response, and developed multiple aquaculture systems. A milestone was reached in 2010 with China’s first fully successful artificial breeding of *P. argenteus*. To date, 1.1 million larvae have been produced and supplied to several coastal provinces [5].

**Figure 5:**
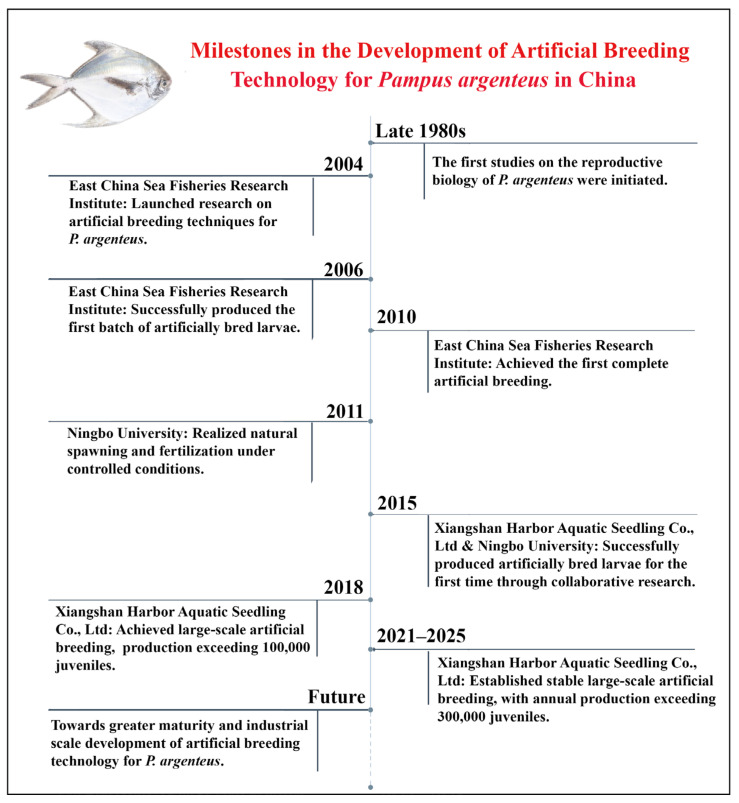
Major milestones in the development of artificial breeding technology for *P. argenteus* in China.

Meanwhile, researchers at Ningbo University achieved a major breakthrough in 2011 by successfully realizing natural fertilization and spawning of *P. argenteus*. Since 2014, the university has collaborated with Xiangshan Harbor Aquatic Seedling Co., Ltd. (Ningbo, China). The company successfully produced its first *P. argenteus* larvae in 2015, with production exceeding 100,000 individuals by 2018. The output reached 418,000 and 350,000 larvae in 2023 and 2024, respectively (company data). Since 2016, larvae from the company have been introduced to various regions, including Zhejiang, Shandong, Jiangsu, Guangdong, Tianjin, as well as offshore aquaculture platforms such as the “Guoxin 101”. The development process of artificial breeding technology for *P. argenteus* in China is shown in Figure 5, and Appendix A shows the status of farming and promotion by major domestic enterprises (or aquaculture platforms).

Although the economic benefits of large-scale aquaculture still require further improvement, critical technical milestones have been achieved. A system for artificial spawning and large-scale larval rearing has been preliminarily established. For industrial aquaculture, a feed supply system and protocols for major disease prevention and monitoring have been developed. Key parameters for cage farming—such as stocking size, density, and management practices—have been standardized. These advances lay a solid foundation for the future large-scale development of *P. argenteus* aquaculture.

Compared with earlier international efforts, the success of *P. argenteus* artificial breeding in China can be attributed to several key innovations. First, breakthroughs in broodstock domestication and stress management significantly improved gamete quality, enabling stable spawning under controlled conditions. Second, refinements in larval feeding protocols—including the sequential use of rotifers, Artemia, and microalgae with optimized nutritional profiles—greatly enhanced early survival rates. Finally, integrated monitoring of water quality allowed for precise management of larvae and juveniles. Collectively, these innovations represent a comprehensive technological framework that has enabled China to achieve full-cycle artificial breeding, where other countries—such as Kuwait—have continued to face persistent constraints in broodstock management, egg quality, and larval survival.

## 5. Current Status of Industrial Technology Development in *P. argenteus*

After over two decades of dedicated research, the *P. argenteus* industry has achieved significant breakthroughs in key areas such as broodstock breeding, artificial propagation, nutritional regulation, and aquaculture systems. While these advancements provide a solid foundation for large-scale industrial development, optimization opportunities remain in certain segments. The specific technological status is detailed below.

### 5.1. Broodstock Cultivation Techniques

The cultivation of *P. argenteus* broodstock has long posed significant technical challenges, primarily due to the species’ extreme susceptibility to scale loss, high stress sensitivity, and consequent mortality. As a result, the direct use of wild-caught broodstock for artificial induction of spawning and reproduction is largely unfeasible. Currently, broodstock are sourced through two main approaches: (1) capture of sexually mature wild individuals, and (2) rearing of artificially produced F1 juveniles or wild-caught fish to maturity under controlled conditions. In early efforts, fertilized eggs were mainly obtained by capturing mature wild fish for artificial fertilization [35,40]. With continued advances in aquaculture techniques, both natural spawning in captivity and fertilized eggs production via hormonal induction have now been achieved domestically and internationally [35,41].

In terms of environmental control, circular tanks with concave bottoms and central drainage are commonly used, but their size is not fixed. Larger water bodies are generally preferred, as they help maintain stable water quality and better support the aquaculture of *P. argenteus*. Water temperature is maintained at 17–19 °C during overwintering via boiler heating and regulated to 25–28 °C during the breeding season. Light intensity is controlled below 700 lx during daytime using shading nets, while water flow is generated by 200–250 W pumps to create circular currents along the tank wall, simulating the natural swimming environment. For nutritional regulation, feed formulations are precisely designed to meet gonadal development needs, containing 42–45% crude protein (fish meal ≥ 40%), 12–15% crude fat (marine fish oil ≥ 8%), and 300–400 mg of vitamin E per kilogram of feed to enhance reproductive performance [5]. Vitamin E, as a potent antioxidant, plays a crucial role in protecting cells from oxidative stress, especially during the energy-intensive processes associated with gonadal development and reproduction. It also supports immune function, which is essential for maintaining overall health and egg quality, and has been shown to improve fertilization rates and larval survival [42].

### 5.2. Artificial Breeding and Larval Rearing Technology

In recent years, artificial breeding technology for *P. argenteus* has evolved from experimental exploration to a systematic process, essentially establishing a complete technical chain covering the “artificial induction of spawning—artificial fertilization—hatching—larval rearing”. Despite breakthroughs in key technological bottlenecks, there remains significant room for improvement in breeding efficiency. Particularly in stress control and early larvae survival rates, which continue to hinder industrial-scale implementation.

Broodstock stress management is critical for successful artificial induction of spawning. During handling, in-water procedures supplemented with appropriate anesthesia should be employed to minimize mechanical stimulation and operational stress. Prior to induction, adding 3–5 mg/L vitamin C to the spawning tank can effectively mitigate stress responses and improve spawning and fertilization rates. A commonly used hormone regimen involves a two-injection protocol of HCG (human chorionic gonadotropin) + LRH-A (luteinizing hormone-releasing hormone analog) + DOM (domperidone) in physiological saline: the first injection delivers 20% of the total dose, followed by the remaining dose 12 h later, administered at the base of the pectoral fin [5].

Broodstock selected for the procedure are typically those whose gonads have reached stage V following hormone induction. Dry fertilization is performed at a 1:1 female-to-male ratio. Within 5–10 min post-fertilization, eggs are rinsed repeatedly with filtered seawater to remove excess sperm and debris. Incubation is conducted in fiberglass conical tanks, with water temperature maintained at 14–18 °C, salinity at 28–33, dissolved oxygen ≥ 6 mg/L, and light intensity ≤ 500 lx. A 30% water exchange is conducted every 6 h, ensuring a temperature differential within 1 °C during each exchange [43]. Fry hatch within 28–36 h [43].

Low survival rates during the larval stage remain the most significant bottleneck in artificial breeding. Rearing conditions must be strictly controlled: salinity 28–34, water temperature 18–24 °C, surface light intensity 500–2000 lx, dissolved oxygen > 5 mg/L, with 5 mg/L EDTA-Na_2_ added to chelate heavy metals. Newly hatched fry are stocked at a density of 15,000–20,000 individuals/m^3^ and transferred together with incubation tank water to the rearing tank. For the first five days, fresh seawater is added daily to raise the water level by 10 cm, or 20–30% water is exchanged, maintaining a depth of about 1.3 m. From day 6 to day 25, 50% of the water is exchanged daily; from day 26 onward, two water exchanges are conducted daily, totaling 100–140% daily. Feeding follows a “staged and diversified” protocol: rotifers (15–20 individuals/mL) are provided initially, followed by *Artemia nauplii* (3–7 individuals/mL), copepods (1–3 individuals/mL), and compound feeds (5–10 particles/mL). Homemade fish paste is used as a supplementary feed, provided 2–4 times daily at amounts consumable within one hour. Bottom siphon cleaning is performed every other day starting from day 3, and daily after day 20. When larvae reach 1.5 cm in length, they are promptly split into separate tanks using water-mediated transfer to minimize stress and scale loss. After 45–50 days of rearing, juveniles with a fork length > 2 cm can be obtained [5].

The brief process of artificial breeding and larval rearing is shown in Table 5.

### 5.3. Nutritional Physiology and Feed Development

Comprehensive feed nutrition is a key factor determining the healthy and rapid growth of farmed *P. argenteus*. A well-balanced feed formulation not only enhances aquaculture production efficiency but also directly impacts profitability. Although no specialized compound feeds are commercially available, extensive research on nutritional physiology has been conducted (Table 6). Peng et al. [44,45] evaluated four dietary treatments and found that fish paste alone did not markedly promote growth in their study, with crude fat content significantly lower than in other groups, suggesting that the dietary fat level may have been below the optimal requirement. Supplementation with copepods not only promoted growth but also enhanced antioxidant capacity. Hossain et al. [36] formulated five isoenergetic and isolipidic diets with protein levels of 35%, 40%, 45%, 50%, and 55%. They found that the optimal protein requirement for juvenile *P. argenteus* (initial body weight: 12.40 ± 0.40 g) was approximately 46–50%. Both excessively low and high protein levels impaired growth and feed utilization. However, this value may vary with fish life stage, environmental conditions, and feed formulation. Zhang [46] further indicated that the substitution of 30% and 70% fish oil with soybean oil slightly enhanced both immunity and antioxidant capacity in juvenile *P. argenteus*.

Previous studies have revealed a distinct feeding preference for jellyfish. After jellyfish consumption, significant increases were observed in the liver concentrations of certain amino acids, amines, and unsaturated fatty acids, alongside enhanced immune function [11,47]. Peng et al. [48,49,50] further investigated the influence of dietary n-3 LC-PUFA levels during vitellogenesis stage on sex steroid hormones, lipid metabolism, and antioxidant capacity. The results showed that moderate n-3 LC-PUFA levels enhanced antioxidant capacity, while high levels significantly regulated lipid metabolism in females by upregulating LPL and downregulating FAS. Li et al. [51] found that dietary supplementation with *Schizochytrium* accelerated growth, improved unsaturated fatty acid content, and enhanced overall health. Additionally, vitamin C and specific probiotics (e.g., *Clostridium butyricum*, *Lactobacillus paraplantarum*, *Bacillus subtilis*) also positively influenced growth and immunity [52,53,54]. Collectively, these studies provide valuable insights into the nutritional physiology of *P. argenteus*, establishing a scientific basis for the development of species-specific, high-performance feeds.

### 5.4. Environmental Physiology and Stress Adaptation Mechanisms

As a eurythermal and euryhaline species, *P. argenteus* can adapt to fluctuations in temperature and salinity but operates within strict tolerance thresholds. Systematically evaluating the effects of environmental factors on its physiology and growth is crucial for optimizing farming practices, reducing aquaculture risks, and enhancing the industrialization process.

Studies have shown that acute temperature stress significantly alters the activity of digestive enzymes, antioxidant enzymes, and metabolic enzymes in juveniles, adversely affecting digestive and metabolic functions [55,56,57]. Liu et al. [58] found that the oxygen consumption rate of juvenile *P. argenteus* was significantly higher at 21 °C and 24 °C than at 27 °C and 30 °C (*p* < 0.05). The Q_10_ value, which represents the sensitivity of fish metabolism to temperature fluctuations and their capacity to adjust metabolic activity following temperature changes, increased from 1.685 in the 21–24 °C to 1.901 in the 24–27 °C, but then decreased to 1.414 in the 27–30 °C. The optimal temperature range for silver pomfret was from 27 to 30 °C in terms of Q_10_ value [58]. Juveniles also exhibit typical osmoregulatory and antioxidant stress responses under salinity variation, maintaining homeostasis by modulating ion-transport enzymes in the gills and kidneys, as well as the antioxidant system in the liver. However, this regulatory response is tissue-specific, and when salinity exceeds the tolerance range, antioxidant capacity and osmoregulatory function become suppressed [59,60,61].

Stocking density critically affects growth, health, and metabolism in aquaculture. Growth rate, glycogen and lactate content, and antioxidant enzyme activity are all influenced by density, with the highest growth rate observed at medium density (approximately 15 ind./m^3^). High density (25 ind./m^3^) may partially decrease antioxidant enzyme activity, but no significant adverse stress response was observed [62]. Additionally, digestive enzyme activity also varies significantly with stocking density, with trypsin, lipase, and amylase activity peaking in the medium-density group, indicating that optimal density enhances both growth and digestive capacity [63]. Ni et al. [64] found that a density of 60 ind./m^3^ promoted growth, while 90 ind./m^3^ increased energy demands, leading to reduced growth rates. Variations in reported optimal densities likely reflect differences in experimental conditions, life stages, and rearing systems.

Live fish transport is a critical link in the aquaculture industry chain. Therefore, researching the physiological regulatory mechanisms under transport stress and identifying optimal transport conditions are essential to minimize stress responses. Peng et al. [65,66] demonstrated that transport stress significantly elevated cortisol, blood glucose, and lactate levels, while mobilizing liver glycogen as the primary energy source. The stress response intensifies with increasing transport density. For 10 g juveniles in small open systems, the recommended maximum transport density is 16 g/L. Both elevated temperature and density reduce survival rates, leading to ammonia nitrogen accumulation and decreased pH. Under high temperature (25 °C) and high density (30–40 g/L), mortality reached 54–64%, whereas at 15 °C, the highest survival rate was maintained even at 40 g/L. Therefore, low temperature combined with low density constitutes the optimal condition for transporting juveniles.

### 5.5. Disease Control and Prevention

The main parasitic threats in *P. argenteus* aquaculture are *Amyloodinium ocellatum* and *Cryptocaryon irritans*. Among these, infection by *A. ocellatum* poses the most severe threat, with the peak incidence occurring at water temperatures of 23–30 °C. This parasite attaches to the gill tissues, causing respiratory obstruction and tissue damage [67], which can lead to mass mortality if not detected promptly. Miao et al. [68] demonstrated that within effective dosage ranges, formaldehyde exhibits lower toxicity, causes less tissue damage, and has a reduced impact on enzyme activity in juveniles compared to copper sulfate. Therefore, formaldehyde should be prioritized, supplemented by freshwater immersion for up to 4 h. *C. irritans* primarily parasitizes the gills, body surface, and fins of *P. argenteus*. Infected fish exhibit symptoms such as restlessness, erratic swimming, isolation from the school, and loss of balance. While copper sulfate and formaldehyde are standard treatments, effective control relies on preventive strategies [69,70,71].

Bacterial diseases are mainly attributed to opportunistic and pathogenic bacteria, particularly *Pseudomonas* and *Photobacterium damselae*, which are commonly associated with fin erosion, congestion, skin ulceration, and body pallor. In addition, infections caused by *Edwardsiella*, *Vibrio alginolyticus*, and certain Pseudomonas species often result in abdominal dropsy, characterized by reduced appetite, mild abdominal distension, serosanguineous fluid accumulation, and fin rot [72,73]. Outbreaks are typically triggered by environmental stressors such as abrupt water quality fluctuations or physical injuries, particularly under elevated temperature and high stocking density. Effective prevention relies on optimized husbandry practices, including appropriate stocking density, superior water quality management, and stress reduction, while therapeutic interventions mainly involve the timely administration of appropriate antibiotics.

Although current studies have characterized the major pathogens and evaluated several chemical and husbandry-based control measures, inconsistencies remain in the reported efficacy of different treatments. For example, variation in the effectiveness of formaldehyde and copper sulfate treatments may arise from differences in fish size, pathogen life stage, exposure duration, and environmental conditions such as temperature and dissolved oxygen. Similarly, disease susceptibility often varies among culture systems and management intensities—recirculating systems with stable water quality typically experience lower infection rates than high-density pond or cage systems.

### 5.6. Innovation and Development of Aquaculture System

Industrial aquaculture is primarily divided into land-based and sea-based systems. Land-based industrial aquaculture includes intensive flow-through culture and recirculating aquaculture systems (RAS). In China, land-based industrial aquaculture originated in the 1960s, initially focused on artificial breeding, and gradually expanded to include the breeding and aquaculture of high-value marine fish. By the early 1990s, it had entered a phase of large-scale development and has since continued to achieve steady progress [74]. In recent years, the ongoing transformation and upgrading of the industry have generated strong demand from enterprises for new industrial aquaculture technologies, which has further accelerated the rapid development of this system. Due to its high stress sensitivity and stringent environmental requirements, *P. argenteus* is currently cultured mainly under industrial aquaculture. Water quality must be maintained within strict ranges: dissolved oxygen > 6 mg/L, temperature 17–32 °C, salinity 20–32, and pH 7.5–8.2, with tight control of ammonia nitrogen and nitrite. A “prevention-first, combined prevention and treatment” approach is adopted, including rational feeding (three times daily, totaling 4–6% of body weight), regular water exchange and bottom cleaning, appropriate stocking density, and periodic tank transfer to reduce disease risks and improve efficiency [5].

Pond aquaculture, the most widely used system in China, generally features lower infrastructure and mechanization compared to industrial aquaculture. Given the strong schooling behavior of *P. argenteus*, traditional pond aquaculture has proven less effective. The common approach now involves placing cages within ponds, emphasizing water disinfection, appropriate stocking density, and enhanced management during critical periods such as high summer temperatures to mitigate stress.

Cage aquaculture has long been the predominant mode of marine fish farming, offering significant advantages in production volume and scale. However, its application for *P. argenteus* has not yielded satisfactory outcomes. In recent years, offshore aquaculture has emerged as a strategic priority promoted by the Chinese government. This system provides advantages such as superior water quality, stable hydrodynamic conditions, and reduced disease risk, holding great potential for industrial application. Although still in the demonstration and pilot promotion stage, it shows promising prospects for large-scale development.

## 6. Bottlenecks in Industrial Development

**Figure 6:**
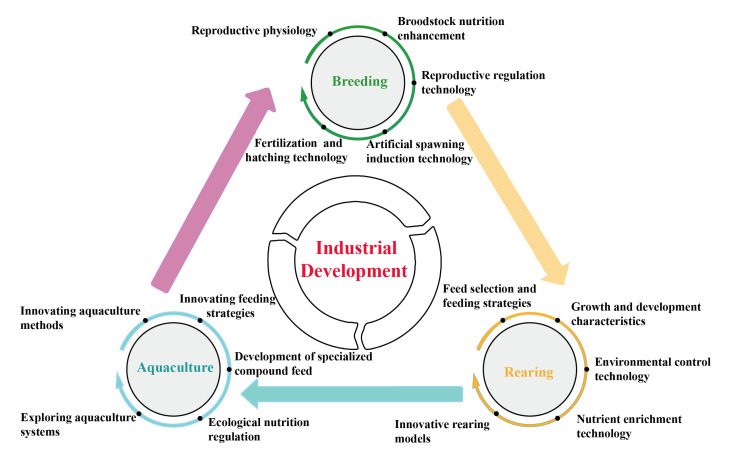
Technical roadmap for the development of the *P. argenteus* aquaculture industry.

The development of the *P. argenteus* aquaculture industry encompasses multiple stages, including broodstock management, fry rearing, and grow-out farming. Despite notable progress in artificial breeding, nutritional regulation, environmental management, and aquaculture systems in recent years, the overall technical framework remains incomplete. Several critical bottlenecks continue to constrain the industry’s transition to large-scale, standardized production (Figure 6). A comprehensive analysis indicates that the major obstacles hindering the sustainable and high-quality growth of the sector are concentrated in five areas: broodstock management, growth performance, feed and nutrition, health control, and the standardization of aquaculture protocols.

### 6.1. Unique Biological Characteristics Lead to Great Difficulties in Aquaculture

Its body is rhombic and laterally compressed, and it lacks a swim bladder. It also has a small, inferior mouth with an immobile lower jaw, and a specialized lateral sac structure at the anterior part of the digestive tract. Its body is covered with delicate cycloid scales that are easily shed, making it highly sensitive to stress and prone to mortality when removed from water. These features collectively contribute to the great challenges encountered in artificial breeding and aquaculture.

### 6.2. Lack of Stable Supply of High-Quality Broodstock

Broodstock quality is a decisive factor influencing breeding efficiency and fry health in *P. argenteus* aquaculture. At present, broodstock are sourced primarily from large wild-caught individuals subsequently domesticated, or hatchery-produced offspring reared and nutritionally fortified for broodstock purposes.

However, wild broodstock are increasingly scarce. Capture operations often cause high rates of injury, and survival during transport and acclimation remains low, resulting in a critical shortage of high-quality broodstock. The current maintenance cost of broodstock for *P. argenteus* is relatively high, and the broodstock individuals are generally small and have low fecundity, with spawning occurring in multiple batches. This makes large-scale and batch production of juveniles difficult to achieve. In addition, the synchronicity of gonadal development between males and females in artificially cultured *P. argenteus* broodstock is poor. These issues not only hinder broodstock renewal and selective breeding programs but also reduce breeding efficiency and undermine the industry’s long-term sustainability.

### 6.3. Slow Growth Rates and Suboptimal Harvest Size

Under aquaculture conditions, *P. argenteus* generally exhibit slow growth and remain relatively small in body size, which significantly reduces farming profitability. The causes are multifactorial, involving both external and internal constraints. Externally, the absence of specialized, high-efficiency feed formulations and optimized culture techniques limits growth potential. Internally, early sexual maturation is prevalent in cultured populations; studies indicate that about 20–40% of individuals initiate gonadal maturation at body weights below 100 g, which subsequently suppresses somatic growth. Moreover, the regulatory mechanisms governing growth traits and genes associated with gonadal development remain poorly characterized, and strategies for genetic improvement and controlling precocious maturation are still in early exploratory stages.

#### 6.3.1. Overall Small Size of Farmed *P. argenteus*

Farmed *P. argenteus* over the course of one year can reach a maximum size of 300–400 g, but large-sized individuals (over 250 g) account for a very small proportion. The small size of farmed *P. argenteus* can generally be attributed to three factors: the broodstock’s genetic quality, the feed used in aquaculture, and the aquaculture system.

#### 6.3.2. Prevalence of Precocious Maturation

Under artificial farming conditions, the minimum size for gonadal maturity in male *P. argenteus* is below 50 g, while in females it is below 100 g. The proportion of precociously matured individuals generally ranges from 20% to 40%.

#### 6.3.3. High Difficulty in Genetic Improvement of Germplasm

Given the current low survival rates during fry rearing (10–20% or lower), limited scale of juvenile production (200,000–300,000 individuals or fewer), and low survival from juvenile to broodstock stage (usually not exceeding 10%), conducting genetic improvement of *P. argenteus* remains highly challenging.

### 6.4. Lagging Development of Specialized Compound Feeds

Although significant progress has been achieved in understanding the nutritional needs of *P. argenteus*, the transition from experimental diets to industrial-scale feed production remains limited. At present, most farms still depend on generic marine fish feeds, leading to low nutrient utilization efficiency and prolonged culture cycles. Several bottlenecks impede feed industrialization: (1) Incomplete nutrient profiling. Research has primarily focused on macronutrients such as protein and lipid, while systematic data on amino acid balance, vitamin–mineral requirements, and energy partitioning remain scarce [46,75,76]. (2) Lack of nutritional modeling. The absence of a precise nutritional database and growth model tailored to *P. argenteus* prevents accurate feed formulation and nutrient optimization. (3) Limited translation of research to practice. Most nutritional studies are small-scale and laboratory-based, with few long-term validation trials under commercial conditions.

### 6.5. Inadequate System for Integrated Disease Control

Although the major pathogens and basic control measures for *P. argenteus* have been identified, the industry still lacks a comprehensive and effective health management system. With increasing stocking densities, disease outbreaks have become more frequent and complex, significantly affecting production stability and profitability. Several major bottlenecks persist: (1) Limited fundamental research. Current studies mainly describe pathogens and treatment outcomes, while systematic investigations into pathogen genomics, infection mechanisms, and host immune responses are scarce. (2) Reliance on empirical medication. Disease control in practice largely depends on experience-based drug use, often without pathogen identification, which increases the risk of antimicrobial resistance and environmental contamination. (3) Insufficient diagnostic and preventive tools. Rapid detection methods, vaccine development, and immunostimulant evaluation remain underdeveloped, hindering early intervention and effective prevention. (4) Weak integration of management and technology. Health management strategies are fragmented across breeding, nutrition, and culture stages, lacking coordination among stakeholders.

### 6.6. Lack of Standardized Aquaculture Protocols

Standardized aquaculture protocols are essential for achieving large-scale production and advancing industry development. At present, no systematic or widely applicable standard operating procedures exist for key stages of *P. argenteus* farming, including broodstock management, seedling production, feed administration, environmental regulation, and disease control. Farming practices largely rely on empirical experience, resulting in considerable operational variability and a lack of unified technical guidelines and quality control standards. In particular, technical parameters for different farming models—such as ponds, land-based intensive systems, and offshore cages—have not been systematically summarized or evaluated. This absence of standardization hinders effective technology transfer and industrial replication, ultimately constraining the orderly and sustainable development of the *P. argenteus* industry.

In summary, we consider that the currently applied stocking densities in *P. argenteus* aquaculture—50–100 individuals per unit water body at the juvenile stage (fork length 3–5 cm) and 15–30 individuals at the large-size stage (fork length over 10 cm)—are generally reasonable. However, recent farming practices indicate that growth performance is still not always ideal. We believe that the main limiting factors are incomplete supporting measures, including feed quality, aquaculture techniques, and disease prevention and control.

## 7. Core Recommendations for Industrial Development

To overcome the key technical bottlenecks currently constraining the *P. argenteus* industry and to facilitate its transition from foundational practices to high-quality development, it is essential to implement a systematic strategy. These include the germplasm conservation and innovation, the cultivation of high-quality broodstock and larvae, the development of specialized feeds, the establishment of robust disease prevention systems, and the optimization of farming practices. Specific countermeasures are outlined as follows.

### 7.1. Elucidate the Mechanisms Underlying Specific Biological Traits and Develop Targeted Aquaculture Techniques

Focusing on the characteristics of *P. argenteus*, such as easily shed scales and high stress sensitivity, in-depth research should be conducted to reveal the regulatory mechanisms underlying these traits. Based on these insights, targeted aquaculture techniques and methods should be developed to reduce stress responses.

### 7.2. Establishment of a Superior Germplasm Resources Base

#### 7.2.1. Systematic Collection and Conservation of Germplasm

Comprehensive surveys should be conducted to assess the geographic distribution and genetic diversity of wild *P. argenteus* populations, with a focus on regions of high genetic value such as the South China Sea and East China Sea. A germplasm repository should be established to preserve populations with diverse genetic backgrounds. High-throughput molecular markers (e.g., single nucleotide polymorphisms, SNPs) should be employed to develop a genetic evaluation system that integrates phenotypic and genotypic data, enabling the identification of strains with superior traits such as fast growth, strong disease resistance, and high flesh quality. Concurrently, artificial domestication and adaptive culture should be implemented, alongside optimized conservation environments and nutritional management, to improve survival and reproductive performance of wild-sourced individuals under controlled conditions.

#### 7.2.2. Construction of Aquatic Original and Improved Strain Breeding Farms

National or provincial-level breeding farms should be established in key aquaculture regions such as Jiangsu, Zhejiang, and Fujian, based on high-quality germplasm. These farms should integrate facilities for germplasm conservation, broodstock selection, reproduction, and grow-out trials. Cooperation with research institutes is crucial to build systems for the long-term preservation and traceable management of germplasm resources. The integration of artificial intelligence and big data technologies will enable refined and intelligent management, ensuring a stable supply of superior larvae for the industry.

### 7.3. Seedling Industry Innovation and Breeding of High-Yield New Varieties

#### 7.3.1. Deciphering the Mechanism of Key Economic Traits

Research should prioritize elucidating the genetic and physiological mechanisms underlying traits that currently limit aquaculture production, such as slow growth, early gonadal development, and high stress sensitivity. A multi-omics approach (encompassing transcriptomics, proteomics, and metabolomics) should be applied to study growth, stress tolerance, and flesh quality. Whole-genome resequencing and Genome-Wide Association Studies (GWAS) can be utilized to identify quantitative trait loci (QTLs) and candidate genes. Subsequent analysis of their expression patterns across different tissues and developmental stages will help reveal the regulatory networks governing these traits.

#### 7.3.2. Creation of Intensive and High-Yield New Varieties

Based on the identification of candidate genes and comprehensive phenotype data, genomic selection (GS) models should be developed to enhance breeding accuracy and efficiency. Family construction and performance evaluation should be conducted in parallel, and germplasm improvement can be accelerated by integrating multi-generation family selection with phenotypic selection strategies. Advanced techniques such as CRISPR/Cas9 may be explored for targeted gene editing to functionally validate key genes, providing a technical foundation for future precision breeding. The ultimate goal is to cultivate new varieties characterized by rapid growth, robust disease resistance, excellent flesh quality, and broad environmental adaptability.

### 7.4. Development of Efficient Artificial Compound Feeds

#### 7.4.1. Building a Comprehensive Nutritional Requirement Database

A systematic nutritional database for *P. argenteus* must be constructed by collecting data on nutrient requirements and physiological responses across different developmental stages. Digestive enzyme activity, intestinal permeability, and microbiota composition should be assessed to evaluate utilization efficiency of proteins, lipids, carbohydrates, and trace elements. Feeding trials under varying environmental conditions (e.g., water temperature, salinity, light) are necessary to refine nutritional models. This database will serve as the scientific foundation for formulating precise and efficient feeds.

#### 7.4.2. Development of Commercial Series of Compound Feeds

Based on the nutritional database, feed formulations should be tailored to the species’ feeding behavior, digestive physiology, and specific aquaculture systems (e.g., cages, ponds, offshore systems). Different specifications of feed should be developed, including floating and sinking varieties, micro-pellets for different life stages, and extruded feeds fortified with functional additives for stress resistance, growth promotion, and immune enhancement. The incorporation of probiotics, enzymes, and immunostimulants can further improve feed efficiency and stock resilience. The industrialization and widespread adoption of these specialized feeds are critical to reducing farming costs and improving overall economic returns.

### 7.5. Establishment of a Precision Disease Prevention and Control System

#### 7.5.1. Analysis of Pathogenic Mechanisms of Aquatic Pathogens

Regular epidemiological surveys should be conducted within aquaculture systems to identify and monitor the prevalence of common pathogens, such as Vibrio and Streptococcus species. Histology, immunology, and molecular methods should be used to reveal pathogenic mechanisms, virulence factors, and host–pathogen interactions. Research should focus on the tropism, replication dynamics, and immune evasion strategies of pathogens within host tissues. This knowledge will contribute to building a holistic “Pathogen-Host-Environment” risk model to explain the high susceptibility and mortality rates observed in culture.

#### 7.5.2. Establishment of an Integrated Prevention and Control Technology System

A comprehensive disease control strategy should adhere to the principle of “prevention first, treatment as a supplement.” Ecological prevention methods include optimizing stocking densities and managing key water quality parameters (e.g., dissolved oxygen, ammonia, pH). For immunological prevention, efforts should focus on developing effective vaccines, including oral and encapsulated formulations suitable for intensive farming, alongside evaluating plant-derived immunostimulants. The use of therapeutics must be standardized through the establishment of clear medication guidelines and residue detection standards, promoting responsible, green, and traceable aquaculture practices.

### 7.6. Establishment of a Standardized Aquaculture Technology System

The whole process of aquaculture should be analyzed to identify key steps and technical parameters. For seedling, standardized evaluation metrics (e.g., body length, health scores) and stocking density guidelines should be established. During the grow-out phase, clear protocols for water quality management, feeding regimes, behavioral observation, and stress assessment are needed. For disease control, a closed-loop system encompassing monitoring, early warning, rapid response, and efficacy evaluation should be institutionalized. Finally, product quality standards—covering sensory, physicochemical, and safety parameters—must be defined, coupled with certification systems for green and high-quality products. The ultimate objective is to form a replicable, standardized technical system covering the entire chain of “seed–culture–management–detection–product,” thereby supporting the sustainable and regulated development of the industry.

## 8. Summary

The development of the *P. argenteus* industry should be guided by an overarching strategy of “breeding-led, technology-driven, model innovation, and industry chain integration,” promoting systematic upgrades across all sectors. Our findings can be summarized as follows: (1) Germplasm as the foundation: Building on the geographical advantages of regions such as Jiangsu, Zhejiang, and Fujian, efforts should focus on collecting and preserving high-quality germplasm from key habitats like the Yellow Sea and East China Sea to support conservation and breeding of new high-yield varieties. (2) Technology as the driver: Strengthening research investment is essential to overcoming bottlenecks in seedling production, feed formulation, and disease control, while accelerating the transfer of practical technologies to farming applications. (3) Model innovation: Diverse aquaculture systems, such as RAS and facility-based pond culture, should be developed and optimized according to local conditions to form regionally distinctive industry clusters. (4) Industry chain integration: Establishing coordinated systems for breeding, culture, feed, processing, and marketing—supported by sound policies and multi-stakeholder collaboration—will facilitate a modern, efficient, and sustainable *P. argenteus* aquaculture ecosystem.

The sustainable development of the *P. argenteus* industry will rely on continuous scientific innovation and technological transformation. Integrating multi-omics research, precision breeding, and intelligent aquaculture systems will deepen our understanding of growth, immunity, and environmental adaptation, thereby enhancing production efficiency and resilience. In parallel, emerging technologies are expected to reshape the field: artificial-intelligence-based monitoring and machine learning tools can enable real-time environmental and health assessment; next-generation vaccine platforms, including recombinant and mRNA-based vaccines, can offer new possibilities for targeted disease prevention; and gene-editing technologies such as CRISPR/Cas present powerful opportunities for improving stress tolerance and feed efficiency. By combining these frontier advances with ecological optimization and industry-chain integration, a modern, intelligent, and sustainable *P. argenteus* aquaculture system can be achieved, driving the high-quality development of China’s marine aquaculture industry.

## Figures and Tables

**Table 1:** Distribution of *Pampus argenteus* [1].

Region	Specific Areas/Countries
Domestic (China)	Bohai Sea, Yellow Sea, East China Sea, and South China Sea
International	Coastal waters from North Korea to Japan
	Bay of Bengal (India)
	Persian Gulf (Iran, Iraq, Kuwait, Saudi Arabia)

**Table 2:** Optimal and tolerance ranges of temperature and salinity for *P. argenteus* under aquaculture conditions [5].

Environmental Factor	Optimal Range	Tolerance Range
Temperature (°C)	25–28	14–32
Salinity (‰)	20–25	10–36

**Table 3:** Statistical table of isozyme band counts in five tissues of *P. argenteus*.

Isozyme	Spleen	Liver	Muscle	Heart	Kidney	Total
EST	1	9	8	9	3	30
LDH	2	5	2	3	4	16
GDH	1	2	1	2	3	9
ADH	1	2	1	1	1	6
Total	5	18	12	15	11	61

**Table 4:** Comparison of overseas progress in artificial breeding of *P. argenteus*.

Country	Research Period	Key Achievements	Larval Survival Rate
Japan	1960s	First artificial insemination and detailed description of embryonic and larval development [30]	/
Kuwait	before 2010	Identified spawning season, fecundity, and reproductive characteristics; improved larval rearing survival from 1.5% to 3.6–4.2%; first successful production from cultured broodstock	3–4% (offspring); 3.6–4.2% (38-day larvae) [31,32,33,34,35]
Kuwait	after 2010	Continued research on broodstock nutrition and management; examined fertilization rate and larval survival	<50% cumulative survival within 24 days post-hatching [36,37,38,39]

**Table 5:** Brief process of artificial breeding and larval rearing [5].

Parameter	Details
Hormone Regimen	2-injection protocol: HCG + LRH-A + DOM (in physiological saline)
Injection Protocol	First injection: 20% of total dose; second injection: remaining 80% after 12 h
Injection Site	Base of the pectoral fin
Female-to-Male Ratio	1:1 (dry fertilization)
Water Quality for Fertilization	Temperature: 14–18 °C, Salinity: 28–33, Dissolved oxygen: ≥6 mg/L, Light: ≤500 lx
Water Quality for Larval Rearing	Temperature: 18–24 °C, Salinity: 28–34, Dissolved oxygen: >5 mg/L, Light: 500–2000 lx
Fry Density	15,000–20,000 individuals/m^3^
Water Exchange (Post-Fry)	50% daily from day 6–25; 100–140% daily from day 26 onward
Feed Protocol	Rotifers (15–20 individuals/mL), *Artemia nauplii* (3–7 individuals/mL), copepods (1–3 individuals/mL), compound feed (5–10 particles/mL)

**Table 6:** Summary of dietary trials and nutritional effects on juvenile *P. argenteus*.

Dietary Treatment	Key Findings
Four Diets (fresh fish meat; fresh fish meat mixed with formulated feed; fresh fish meat mixed with formulated feed and solen meat; fresh fish meat mixed with formulated feed, solen meat and copepods)	Supplementation with copepods promoted growth [44,45]
35%, 40%, 45%, 50%, 55% protein diets	The optimal protein content is 46–50% [36]
Four Diets (100% fish oil; 70% fish oil and 30% soybean oil; 30% fish oil and 70% soybean oil; 100% soybean oil)	Replacing fish oil with soybean oil (18–24% n-3 LC-PUFA, balanced n-3/n-6 ratio of 1–2) promoted healthy growth [46]
Adding Jellyfish to the Diet	Increased liver amino acids, amines, and unsaturated fatty acids [11,47]
n-3 LC-PUFA levels during vitellogenesis	Enhanced antioxidant capacity, lipid metabolism regulation [48,49,50]
*Schizochytrium* Supplementation	Accelerated growth, improved unsaturated fatty acids [51]
Vitamin C and Probiotics	Positive effects on growth and immunity [52,53,54]

## Data Availability

No new data were created or analyzed in this study.

## Appendix A. Status of Farming and Promotion by Major Domestic Enterprises (or Aquaculture Platforms)

1. Xiangshan Harbor Aquatic Seed Co., Ltd. (Ningbo, China).

Since 2014, Xiangshan Harbor Aquatic Seed Co., Ltd. (Ningbo, China). has collaborated with Ningbo University on artificial breeding and aquaculture research of *P. argenteus*. In 2015, the company successfully produced *P. argenteus* juveniles for the first time. By 2018, the number of juveniles produced exceeded 100,000 for the first time. In 2019, the breeding operations achieved production based on market demand, and the number of juveniles steadily increased. In 2023 and 2024, the output remained stable at over 400,000 juveniles. Since 2016, *P. argenteus* juveniles have been gradually promoted to Zhejiang, Shandong, Jiangsu, Guangdong, Tianjin, and offshore aquaculture platforms such as Guoxin Industrial Vessel. However, due to the species’ unique biological characteristics, farming remains challenging, and the actual economic benefits of promotional aquaculture are still limited. Currently, large-scale aquaculture technology for *P. argenteus* is not yet fully mature. Nevertheless, after nearly ten years of research and development, Xiangshan Harbor Aquatic Seed Co., Ltd. has accumulated substantial practical experience in breeding and aquaculture, initially establishing a technical system for artificial induction and large-scale seed production, and developing a complete set of feed management and major disease prevention and monitoring techniques under factory-based aquaculture. Key technologies for cage aquaculture, including stocking size, density, and management procedures, have been mastered, providing strong support for the future large-scale development of the *P. argenteus* aquaculture industry.

2. Ningbo Yuanfang Technology Co., Ltd. (Ningbo, China).

The juveniles used for aquaculture are sourced from Xiangshan Harbor Aquatic Seed Co., Ltd. (Ningbo, China). In 2024, the company purchased 60,000 juveniles larger than 3 cm and cultured them using RAS. Growth rates were significantly faster than in traditional system, and the survival rate has remained high.

3. Guoxin 101

In 2023, 200,000 *P. argenteus* juveniles were purchased from Xiangshan Harbor Aquatic Seed Co., Ltd. During the initial stage, juveniles were cultured in netted enclosures within the tanks, with satisfactory results. However, mortality increased when the net cages were removed, resulting in an unsuccessful trial. In 2024, 120,000 juveniles were purchased from Xiangshan Harbor Aquatic Seed Co., Ltd., and the farming progress is currently proceeding smoothly.

4. Donghai No.1

In mid-October 2025, approximately 10,000 large-sized juveniles (approximately 20 g each) were purchased from Xiangshan Harbor Aquatic Seed Co., Ltd. to conduct a relay-style aquaculture experiment.
